# Incidence of Parkinson’s disease and modifiable risk factors in Korean population: A longitudinal follow-up study of a nationwide cohort

**DOI:** 10.3389/fnagi.2023.1094778

**Published:** 2023-02-14

**Authors:** Sung Hoon Kang, Seok-Joo Moon, Minwoong Kang, Su Jin Chung, Geum Joon Cho, Seong-Beom Koh

**Affiliations:** ^1^Department of Neurology, Korea University Guro Hospital, Korea University College of Medicine, Seoul, Republic of Korea; ^2^Smart Healthcare Center, Korea University Guro Hospital, Korea University College of Medicine, Seoul, Republic of Korea; ^3^Department of Biomedical Research Center, Korea University Guro Hospital, Korea University College of Medicine, Seoul, Republic of Korea; ^4^Department of Neurology, Myongji Hospital, Hanyang University College of Medicine, Goyang, Republic of Korea; ^5^Department of Obstetrics and Gynecology, Korea University Guro Hospital, Korea University College of Medicine, Seoul, Republic of Korea

**Keywords:** Parkinson’s disease, incidence, modifiable risk factor, cardiometabolic syndrome, osteoporosis, depression

## Abstract

**Introduction:**

We aimed to investigate the incidence of Parkinson’s disease (PD) by age and year for each sex as well as the modifiable risk factors for PD. Using data from the Korean National Health Insurance Service, 938,635 PD and dementia-free participants aged ≥40 years who underwent general health examinations were followed to December 2019.

**Methods:**

We analyzed the PD incidence rates according to age, year and sex. To investigate the modifiable risk factors for PD, we used the Cox regression model. Additionally, we calculated the population-attributable fraction to measure the impact of the risk factors on PD.

**Results:**

During follow-up, 9,924 of the 938,635 (1.1%) participants developed PD. The incidence of PD increased continuously from 2007 to 2018, reaching 1.34 per 1,000 person-years in 2018. The incidence of PD also increases with age, up to 80 y. Presence of hypertension (SHR = 1.09, 95% CI 1.05 to 1.14), diabetes (SHR = 1.24, 95% CI 1.17 to 1.31), dyslipidemia (SHR = 1.12, 95% CI 1.07 to 1.18), ischemic stroke (SHR = 1.26, 95% CI 1.17 to 1.36), hemorrhagic stroke (SHR = 1.26, 95% CI 1.08 to 1.47), ischemic heart disease (SHR = 1.09, 95% CI 1.02 to 1.17), depression (SHR = 1.61, 95% CI 1.53 to 1.69), osteoporosis (SHR = 1.24, 95% CI 1.18 to 1.30), and obesity (SHR = 1.06, 95% CI 1.01 to 1.10) were independently associated with a higher risk for PD.

**Discussion:**

Our results highlight the effect of modifiable risk factors for PD in the Korean population, which will help establish health care policies to prevent the development of PD.

## 1. Introduction

As the population ages, the number of patients with neurodegenerative diseases also rapidly increase, along with an increased socioeconomic burden (Bach et al., 2011). Parkinson’s disease (PD) is the second most common neurodegenerative disease and is characterized by progressive non-motor symptoms and motor deficits, including bradykinesia, tremors, and rigidity. Given that PD currently has no disease-modifying treatment, it is important to identify the incidence and modifiable risk factors of PD and to find effective strategies for preventing PD in public health care policies.

The incidence of PD varies across countries, ranging from 80.4 to 678 per 100,000 person-years (Baldereschi et al., 2000; Benito-León et al., 2004; de Lau et al., 2004; Taylor et al., 2006; Alves et al., 2009; Driver et al., 2009; Linder et al., 2010; Winter et al., 2010; Caslake et al., 2013). In the Korean population, the prevalence of PD has been steadily increasing, and the prevalence in people aged ≥50 y is approximately 0.4% (Park J. H. et al., 2019). However, a detailed information regarding age-specific PD incidence is lacking.

A variety of known modifiable risk factors exist for PD with varying degrees of impact on PD (Ascherio and Schwarzschild, 2016). However, the extent to which modifiable risk factors affect PD remains controversial. The prevention of PD has been the focus of research owing to the absence of disease-modifying medications. Risk factors that have a higher relative risk (RR) for PD and a higher prevalence in the elderly population may contribute more to PD incidence. Therefore, the RR and prevalence of each risk factor in the elderly population should be considered to establish public health care measures for PD prevention. In addition, among the risk factors, the effect of cardiometabolic syndrome on PD risk has not been established, although a growing body of evidence has shown that cardiometabolic syndrome is closely related to Alzheimer’s disease, included in neurodegenerative disease with PD.

Using the Korean National Health Insurance Service (KNHIS) data, the first goal of our study was to investigate PD incidence by age and year for each sex. The second goal was to explore the hazard ratio (HR) of each modifiable risk factor for PD. The third goal was to evaluate the RR of each modifiable risk factor for PD and estimate the attributable fraction of the risk factors in elderly Koreans.

## 2. Materials and methods

This study was approved by the Institutional Review Board of the Korea University Guro Hospital and adhered to the principles of the Declaration of Helsinki.

### 2.1. Data source

We used a customized dataset from the KNHIS, which includes more than 99% of the Korean population (approximately 50 million).^1^ The KNHIS database includes personal information; health insurance claim codes (procedures and prescriptions); diagnostic codes from the Korean Standard Classification of Diseases, 7th Revision, which is based on the International Classification of Diseases, 10th Revision (ICD-10); death records from the Korean National Statistical Office; and general medical examination data for each participant from 2002 to 2019. Data on body mass index (BMI) and behavioral characteristics, including frequency of physical activity, smoking, and alcohol consumption, were obtained from the general health examinations in the KNHIS database.

### 2.2. PD and dementia-free cohort

To exclude participants with PD and dementia, PD was defined according to the ICD-10 code (G20) and prescriptions of PD medication. Dementia was defined according to the ICD-10 codes (F00, F01, F02, F03, F05, G30, or G31) and dementia medication prescription.

In the NHIS dataset, 6,257,567 PD and dementia-free participants aged 45 y or older who underwent general health examinations were identified. We randomly selected 15% (938,635) of the participants and enrolled them in the present study.

### 2.3. Definition of modifiable risk factors

With respect to the modifiable risk factors for PD, we considered hypertension, diabetes, hyperlipidemia, ischemic stroke, hemorrhagic stroke, ischemic heart disease, depression, osteoporosis, obesity, physical inactivity, smoking status, and heavy alcohol consumption. The presence of hypertension was defined according to ICD-10 code (I10-15) and prescription of antihypertensive medication. The presence of diabetes was defined according to the ICD-10 code (E8-14) and prescription of antidiabetic medication. The presence of hyperlipidemia was defined according to the ICD-10 code (E78) and the prescription of lipid-lowering medication. The presence of ischemic stroke was defined according to the ICD-10 code (I63-66) and prescription of antiplatelet or anticoagulation agents. Hemorrhagic stroke was defined according to the ICD-10 code (I60-62). The presence of ischemic heart disease was defined according to the ICD-10 code (I20-25) and the prescription of antiplatelet or anticoagulation agents. Depression was defined according to the ICD-10 code (F32-34). Osteoporosis was defined according to the ICD-10 code (M80-82). Obesity was defined as a BMI ≥ 25 kg/m^2^. Physical inactivity was defined as the absence of physical activity, even once a week. Smoking status was grouped into three levels: never smoked, ex-smoker, and current smoker. Heavy alcohol consumption was defined as alcohol consumption more than three times per week.

### 2.4. Definition of outcome and follow-Up

The outcome of the study was the development of PD, which was defined according to the ICD-10 code (G20) and prescription of PD medication for ≥3 months. Furthermore, to exclude secondary parkinsonism and atypical parkinsonism, such as progressive supranuclear palsy and multiple system atrophy, we excluded participants who additionally had ICD-10 code (G21-23) after diagnosis of PD from the outcome. Participants without PD during follow-up were considered to have completed the study on the date of death or at the end of follow-up. The patients were followed up from the date of the general health examinations (baseline) to the date of PD diagnosis, date of death, or until December 2019.

### 2.5. Statistical analyses

Baseline characteristics are presented as mean ± standard deviation or median (interquartile range) and frequency (%). First, in the PD-free cohort, we calculated the PD incidence rates and confidence intervals of the incidence rates under the assumption that the number of outcomes follows a Poisson distribution. Second, to investigate the modifiable risk factors for PD, we calculated HR using the Cox regression model, including each modifiable risk factor as a separate predictor after controlling for age and sex (model 1). We further performed the Cox regression model including modifiable risk factors as predictors that showed statistical significance in model 1 after controlling for age and sex (model 2). Third, we calculated the RR using log-binomial regression to adjust for age, sex, and modifiable risk factors (McNutt et al., 2003). These models included the risk factors that showed statistical significance in Cox regression model 1 and were controlled for age and sex. Finally, the population-attributable fraction (PAF) was calculated using Levin’s formula:


$$PAF=\frac{P_{\mathrm{risk}}\times \left(RR-1\right)}{1+P_{\mathrm{risk}}\times \left(RR-1\right)}\text{.}$$

With respect to the prevalence of modifiable risk factors, we considered the prevalence in our cohort as presented in Table 1. To identify the combined effects of risk factors, we obtained the overall PAFs using the following formula:


$$\mathrm{Overall}\;PAF=1-\left(1-PAF_{1}\right)\times \left(1-PAF_{2}\right)\times \left(1-PAF_{3}\right)\times \ldots$$

Sensitivity analyses were used to exclude participants with stroke to eliminate the mediating or confounding effects of stroke on the relationship between the modifiable risk factors and the development of PD.

**Table 1:** Baseline characteristics of the study participants.

Characteristics at initial visit	Total (*n =* 938,635)	PD-free at follow-up (*n =* 928,711)	Incident PD at follow-up (*n =* 9,924)
**Sex**
Male (%)	475,609	471,207 (50.7%)	4,402 (44.4%)
Female (%)	463,026	457,504 (49.3%)	5,522 (55.6%)
**Age groups, y**
≤50	155,240	154,895 (16.7%)	345 (3.5%)
51–55	216,621	215,862 (23.2%)	759 (7.7%)
56–60	169,606	168,432 (18.1%)	1,174 (11.8%)
61–65	138,092	136,329 (14.7%)	1,763 (17.8%)
66–70	121,046	118,542 (12.8%)	2,504 (25.2%)
71–75	77,919	75,890 (8.2%)	2,029 (20.5%)
76–80	38,910	37,890 (4.1%)	1,020 (10.3%)
81–85	15,687	15,407 (1.7%)	280 (2.8%)
≥86	5,514	5,464 (0.6%)	50 (0.5%)
Hypertension (%)	344,839	339,625 (36.6%)	5,214 (52.5%)
Diabetes (%)	97,162	95,514 (10.3%)	1,648 (16.6%)
Dyslipidemia (%)	139,135	137,049 (14.8%)	2,086 (21.0%)
Ischemic stroke (%)	33,408	32,576 (3.5%)	832 (8.4%)
Hemorrhagic stroke (%)	8,538	8,362 (0.9%)	176 (1.8%)
Ischemic heart disease (%)	92,466	53,126 (5.7%)	1,039 (10.5%)
Depression (%)	107,609	105,493 (11.36%)	2,116 (21.3%)
Osteoporosis (%)	199,675	196,332 (21.1%)	3,343 (33.7%)
Obesity (%)	340,780	337,065 (36.3%)	3,715 (37.4%)
Physical inactivity (%)	513,526	507,495 (54.7%)	6,031 (60.8%)
Heavy alcohol consumption (%)	100,300	99,374 (10.7%)	926 (9.3%)
Smoking			
Never	672,659	664,801 (71.6%)	7,858 (79.2%)
Ex-smoker	86,394	85,671 (9.2%)	723 (7.3%)
Current smoker	179,582	178,239 (19.2%)	1,342 (13.5%)

PD, Parkinson’s disease.

All reported *p*-values were two-sided and the significance level was set at 0.05. All analyses were performed using SAS (version 9.3; SAS Institute Inc., Cary, NC, United States).

## 3. Results

### 3.1. Clinical characteristics of the study participants at baseline

Among the 938,635 participants in the PD and dementia-free cohorts, 463,026 (49.3%) were women. The most prevalent modifiable risk factor was physical inactivity (54.7%), followed by hypertension (36.7%), obesity (36.3%), osteoporosis (21.3%), and current smoking (19.1%, Table 1). Subjects who developed PD were more likely to be and have hypertension, diabetes, dyslipidemia, ischemic heart disease, ischemic stroke, hemorrhagic stroke, depression, and osteoporosis than those who were PD-free at follow-up.

### 3.2. PD incidence by year, age, and sex

During follow-up, 9,924 of the 938,635 (1.1%) participants developed PD. The incidence rate of PD showed annual growth, increasing from 0.56 per 1,000 person-years in 2006 to 1.34 per 1,000 person-years in 2018 (Table 2). The incidence rate of PD also increases with age, up to 80 y. Specifically, the incidence rate of PD was only 0.19 per 1,000 person-years among participants who were 50 y and less, while that of PD increased to 2.91 per 1,000 person-years among participants who were 76 to 80 y old (Table 3). In terms of sex, women (9,447; 54.4%) were more likely to develop PD than men (7,910; 45.57%). The incidence rate of PD in women (1.05 per 1,000 person-years) was higher than that in men (0.84 per 1,000 person-years, Table 3).

**Table 2:** PD incidence by year.

	Number of person-years			Number of *de novo* PD cases		PD incidence rate (95% CI) (per 1,000 person-years)			
	Total	M	F	Total	M	F	Total	M	F
2007	934139.8	472710.1	461429.7	527	253	274	0.56 (0.52–0.61)	0.54 (0.47–0.61)	0.59 (0.53–0.67)
2008	925348.4	466986.5	458361.9	607	240	367	0.66 (0.61–0.71)	0.51 (0.45–0.58)	0.80 (0.72–0.89)
2009	916,141	461085.9	455055.1	648	290	358	0.71 (0.65–0.76)	0.63 (0.56–0.71)	0.79 (0.71–0.87)
2010	906457.9	454953.1	451504.8	785	328	457	0.87 (0.81–0.93)	0.72 (0.65–0.80)	1.01 (0.92–1.11)
2011	896023.2	448534.3	447488.9	713	305	408	0.80 (0.74–0.86)	0.68 (0.61–0.76)	0.91 (0.83–1.00)
2012	885027.9	441688.8	443339.1	826	356	470	0.93 (0.87–1.00)	0.81 (0.73–0.89)	1.06 (0.97–1.16)
2013	873650.3	434776.8	438873.5	897	400	497	1.03 (0.96–1.10)	0.92 (0.83–1.01)	1.13 (1.04–1.24)
2014	861964.1	427688.7	434275.4	842	375	467	0.98 (0.91–1.05)	0.88 (0.79–0.97)	1.08 (0.98–1.18)
2015	849620.8	420361.7	429259.1	919	428	491	1.08 (1.01–1.15)	1.01 (0.93–1.12)	1.14 (1.05–1.25)
2016	836753.7	412908.7	423,845	1,022	445	577	1.22 (1.15–1.30)	1.08 (0.98–1.18)	1.36 (1.25–1.48)
2017	823391.4	405168.8	418222.6	1,053	487	566	1.28 (1.20–1.36)	1.20 (1.10–1.31)	1.35 (1.25–1.47)
2018	808850.4	396820.9	412029.5	1,085	495	590	1.34 (1.26–1.42)	1.25 (1.14–1.36)	1.43 (1.32–1.55)

CI, confidence interval; PD, Parkinson’s disease.

**Table 3:** PD incidence by age.

Number of person-years			Number of *de novo* PD cases		PD incidence rate (95% CI)* (per 1,000 person-years)			
Total	M	F	Total	M	F	Total	M	F
10,496,949	5237349.6	5259599.1	9,924	4,402	5,522	0.95 (0.93–0.96)	0.84 (0.82–0.87)	1.05 (1.02–1.08)
1,826,541	1020941.4	805599.6	345	193	152	0.19 (0.17–0.21)	0.19 (0.16–0.22)	0.19 (0.16–0.22)
2534288.7	1312161.9	1222126.8	759	366	393	0.30 (0.28–0.32)	0.28 (0.25–0.31)	0.32 (0.29–0.36)
1961058.7	992969.9	968088.8	1,174	574	600	0.60 (0.57–0.63)	0.58 (0.53–0.63)	0.62 (0.57–0.67)
1564566.1	767917.7	796648.4	1763	760	1,003	1.13 (1.08–1.18)	0.99 (0.92–1.06)	1.26 (1.18–1.34)
1319382.1	611688.7	707693.4	2,504	1,067	1,437	1.90 (1.83–1.97)	1.74 (1.64–1.85)	2.03 (1.93–2.14)
793203.5	343,126	450077.5	2029	853	1,176	2.56 (2.45–2.67)	2.49 (2.32–2.66)	2.61 (2.47–2.77)
350548.8	133,240	217308.8	1,020	449	571	2.91 (2.74–3.09)	3.37 (3.07–3.70)	2.63 (2.42–2.85)
116073.6	43786.3	72287.3	280	119	161	2.41 (2.15–2.71)	2.72 (2.27–3.25)	2.23 (1.91–2.60)
31286.2	11517.7	19768.5	50	21	29	1.60 (1.21–2.11)	1.82 (1.19–2.80)	1.47 (1.02–2.11)

^*^CI of the incidence rate was obtained under the assumption that the number of events follows a Poisson distribution.

CI, confidence interval; PD, Parkinson’s disease.

### 3.3. Modifiable risk factors for PD

In model 1, presence of hypertension (subdistribution hazard ratio [SHR] = 1.27, 95% confidence interval [CI] 1.22 to 1.33), diabetes (SHR = 1.39, 95% CI 1.32 to 1.47), dyslipidemia (SHR = 1.34, 95% CI 1.28 to 1.41), ischemic stroke (SHR = 1.58, 95% CI 1.47 to 1.70), hemorrhagic stroke (SHR = 1.54, 95% CI 1.32 to 1.78), ischemic heart disease (SHR = 1.38, 95% CI 1.30 to 1.47), depression (SHR = 1.76, 95% CI 1.68 to 1.85), osteoporosis (SHR = 1.34, 95% CI 1.28 to 1.41), and obesity (SHR = 1.12, 95% CI 1.07 to 1.16) increased the risk of PD (Table 4). In model 2, presence of hypertension (SHR = 1.09, 95% CI 1.05 to 1.14), diabetes (SHR = 1.24, 95% CI 1.17 to 1.31), dyslipidemia (SHR = 1.12, 95% CI 1.07 to 1.18), ischemic stroke (SHR = 1.26, 95% CI 1.17 to 1.36), hemorrhagic stroke (SHR = 1.26, 95% CI 1.08 to 1.47), ischemic heart disease (SHR = 1.09, 95% CI 1.02 to 1.17), depression (SHR = 1.61, 95% CI 1.53 to 1.69), osteoporosis (SHR = 1.24, 95% CI 1.18 to 1.30), and obesity (SHR = 1.06, 95% CI 1.01 to 1.10) remained independently associated with a higher risk for PD (Table 4).

**Table 4:** Hazard ratio of modifiable risk factors for PD.

	Model 1*		Model 2^#^	
	HR (95% CI)	*p*	HR^#^ (95% CI)	*p*
Hypertension	1.27 (1.22–1.33)	<0.001	1.09 (1.05–1.14)	<0.001
Diabetes	1.39 (1.32–1.47)	<0.001	1.24 (1.17–1.31)	<0.001
Dyslipidemia	1.34 (1.28–1.41)	<0.001	1.12 (1.07–1.18)	<0.001
Ischemic stroke	1.58 (1.47–1.70)	<0.001	1.26 (1.17–1.36)	<0.001
Hemorrhagic stroke	1.54 (1.32–1.78)	<0.001	1.26 (1.08–1.47)	0.003
Ischemic heart disease	1.38 (1.30–1.47)	<0.001	1.09 (1.02–1.17)	0.012
Depression	1.76 (1.68–1.85)	<0.001	1.61 (1.53–1.69)	<0.001
Osteoporosis	1.34 (1.28–1.41)	<0.001	1.24 (1.18–1.30)	<0.001
Obesity	1.12 (1.07–1.16)	<0.001	1.06 (1.01–1.10)	0.011
Physical inactivity	0.99 (0.95–1.03)	0.544		
Heavy alcohol consumption	0.96 (0.89–1.03)	0.247		
**Smoking**
Never	Reference		Reference	
Ex-smoker	0.82 (0.76–0.89)	<0.001	0.82 (0.76–0.89)	<0.001
Current smoker	0.76 (0.74–0.83)	<0.001	0.81 (0.76–0.86)	<0.001

^*^Model 1: Each risk factor was entered as a predictor in the Cox regression model after controlling for age and sex.

# Model 2: All risk factors associated with PD in model 1 were included as predictors after controlling for age and sex.

HR, hazard Ratio; PD, Parkinson’s disease.

### 3.4. Population-attributable fraction for PD

As presented in Table 5, among the modifiable risk factors, depression had the greatest impact on PD (PAF, 6.5%), followed by osteoporosis (PAF, 4.8%), and hypertension (PAF, 3.3%). The overall PAF of the modifiable risk factors was 20.4%.

**Table 5:** Relative risk and population-attributable fraction of modifiable risk factors for PD.

	Risk factor prevalence	Parkinson’s disease	
		Relative risk* (95% CI)	PAF (95% CI)
Hypertension	36.7%	1.09 (1.05–1.14)	3.3% (1.6–5.0)
Diabetes	10.4%	1.24 (1.17–1.31)	2.4% (1.8–3.1)
Dyslipidemia	14.8%	1.12 (1.07–1.18)	1.8% (1.0–2.6)
Ischemic stroke	3.6%	1.26 (1.17–1.36)	0.9% (0.6–1.3)
Hemorrhagic stroke	0.9%	1.26 (1.08–1.47)	0.2% (0.1–0.4)
Ischemic heart disease	5.8%	1.09 (1.02–1.17)	0.5% (0.1–1.0)
Depression	11.5%	1.61 (1.53–1.69)	6.5% (5.7–7.3)
Osteoporosis	21.3%	1.24 (1.18–1.30)	4.8% (3.7–6.0)
Obesity	36.3%	1.06 (1.01–1.10)	2.0% (0.5–3.5)
Overall PAF			20.4%

^*^RR was calculated after controlling for age, sex, and all the risk factors associated with PD.

CI, confidence interval; PD, Parkinson’s disease.

### 3.5. Sensitivity analyses

Among the participants without stroke, hypertension (SHR = 1.09, 95% CI 1.03 to 1.13), diabetes (SHR = 1.24, 95% CI 1.17 to 1.32), dyslipidemia (SHR = 1.14, 95% CI 1.08 to 1.21), ischemic heart disease (SHR = 1.13, 95% CI 1.04 to 1.22), depression (SHR = 1.68, 95% CI 1.59 to 1.77), osteoporosis (SHR = 1.25, 95% CI 1.19 to 1.31), and obesity (SHR = 1.05, 95% CI 1.00 to 1.10) were independently associated with a higher risk for PD (Supplementary Table 1).

## 4. Discussion

In the present study, we identified the incidence and modifiable risk factors of PD using the Korean nationwide cohort data. The major findings of this study are as follows. First, the incidence of PD increased continuously from 2007 to 2018, reaching 1.34 per 1,000 person-years in 2018. Second, the incidence of PD increases with age, up to 80 y. Third, cardiometabolic syndromes, depression, and osteoporosis are associated with a higher incidence of PD, independent of stroke. Overall, our results will help in the design of public health policies for PD prevention.

Our first major finding was the increasing trend in the incidence of PD in South Korea from 2007 to 2018. Trends in PD incidence vary depending on the study design, population, and period. Stable or slightly decreasing trends have been reported in Western countries, such as the United States, the United Kingdom, France, and the Netherlands during the 2010s (Akushevich et al., 2013; Horsfall et al., 2013; Blin et al., 2015; Darweesh et al., 2016; Evans et al., 2016). Conversely, several studies have reported an annual increase in PD incidence (Liu et al., 2016; Savica et al., 2016). In the Minnesota population aged ≥70 years old and older, the incidence of PD increased from 0.80 per 1,000 person-years to 1.37 per 1,000 person-years over 30 y (Savica et al., 2016). An increasing trend has also been identified in Taiwan, which is included in the far-eastern Asian countries along with Korea (Liu et al., 2016). This increasing trend may be attributed to better recognition of PD in older patients with comorbidities. In recent years, physicians have begun to diagnose elderly individuals with cancer, cardiovascular diseases, or other conditions as having PD, because parkinsonism symptoms have become important in the overall clinical outcome and are considered one of the major causes of disability and mortality. In addition, the KNHIS started to cover dopamine transporter images in 2016, and consequently, the diagnosis of PD became relatively simplified, which could contribute to an increasing point of PD incidence in 2016. The increasing incidence of PD may also be explained by the increase in the prevalence of modifiable risk factors for PD, such as hypertension and dyslipidemia, and the dramatic decrease in the rate of smoking, a protective factor for PD, in Korea (Korea Health Statistics 2019, Korea National Health and Nutrition Examination Survey,^2^).

Our second major finding was that the incidence of PD increased with age, up to 80 y. It is well known that PD prevalence is low (0.13–1.6%) in populations aged less than 60 y, after which there is a sharp increase in incidence (Kis et al., 2002; Benito-León et al., 2003; Chan et al., 2005; Blin et al., 2015). These results are consistent with our findings that PD incidence was only 0.36 per 1,000 person-years among participants aged 60 y or less, whereas PD incidence increased to 2.91 per 1,000 person-years among those aged 76–80 y. Although age may be an important risk factor for PD, peak age-specific incidence varies among studies. Several studies have reported that PD incidence uniformly increases up to the ninth decade (Allyson Jones et al., 2012; Caslake et al., 2013; Blin et al., 2015), whereas others have found a decline in PD incidence among the oldest old population. We also found that the age-specific incidence peaked in the population aged 76–80 y and declined beyond this age. However, a direct comparison between the studies is difficult because of the small sample sizes in the oldest old group and varying definitions (von Campenhausen et al., 2005). The decline in the oldest old population may be due to the following reasons. First, the high burden of comorbidities, such as dementia and musculoskeletal disease, in the oldest old group increases the diagnostic uncertainty for PD (Meara et al., 1999). Second, individuals with PD at the oldest old age may not use a medical institution (Bowling et al., 1991). Third, mortality selection may cause unobserved heterogeneity within the oldest old group, which in turn determines the ratio of individuals with and without PD in favor of the latter group (Vaupel et al., 1979).

Contrary to our expectations, we observed that PD incidence in women was significantly higher than that in men. A growing body of evidence shows a predominance of PD incidence in the male population (Baldereschi et al., 2000; Clavería et al., 2002; Benito-León et al., 2003; Alves et al., 2009; Nerius et al., 2017) or no sex difference in PD incidence (Linder et al., 2010; Winter et al., 2010). However, a few Asian studies have reported female predominance in PD (Kimura et al., 2002; Park J. H. et al., 2019). Although the underlying mechanisms for this discrepancy remain unclear, genetic, hormonal, cultural, and environmental factors may mediate such outcomes. First, Asian women may have different genetic susceptibilities to PD. Second, substantial differences in cultural and environmental factors during childhood in the elderly population (in the mid 1900s) in Korea may cause sex-related disparities in educational levels and literacy rates associated with brain reserves. Furthermore, Korean women may encounter more risk factors, including dietary deficiencies, agricultural occupations, pesticide use, and head trauma. Finally, the greater average longevity of women in Korea may lead to a quicker increase in the elderly female population, which in turn causes female predominance. In fact, the sex-specific difference in life expectancy was higher in South Korea (men, 79.7 y and women, 85.7 y) than in European countries (men, 79.7 y and women, 82.8 y) according to the Office for National Statistics.

Our third major finding was that cardiometabolic syndrome, depression, and osteoporosis were associated with a higher incidence of PD, independent of stroke. In particular, among cardiometabolic syndromes, diabetes contributes the most to the development of PD, which is consistent with previous findings that diabetes is associated with a higher PD risk (Hu et al., 2007; Driver et al., 2008; Schernhammer et al., 2011; Xu et al., 2011; Sun et al., 2012). However, the association between hypertension, dyslipidemia, obesity, and PD remains controversial. Many previous studies in Western countries have shown that hypertension, dyslipidemia, and obesity are not associated with a higher risk of PD (Abbott et al., 2002; Logroscino et al., 2007; Simon et al., 2007; Kyrozis et al., 2013; Ascherio and Schwarzschild, 2016), whereas a few studies have reported that dyslipidemia and obesity may be risk factors for PD (Hu et al., 2006, 2008). These discordant results may be related to the modest effect of cardiometabolic syndrome on incident PD, or undiscerned confounding or modifying factors that modulate the relationship between cardiometabolic syndrome and PD risk. Another explanation for this discrepancy is ethnic differences in the effects of cardiometabolic syndromes on PD. Compared to European populations, Asian populations have a higher incidence of cardiometabolic syndrome (Yoon et al., 2006) and associated complications, including coronary artery disease (McKeigue et al., 1989), stroke (Eastwood et al., 2015), dementia (Niu et al., 2017; Park J. E. et al., 2019; Jang et al., 2021), and high mortality rates (Wild et al., 2007). Additionally, studies have shown that Asian populations tend to have higher visceral fat and lower subcutaneous fat than European populations with similar BMI (Nazare et al., 2012). Such unequal fat distribution may be associated with more severe cardiometabolic complications commonly seen in Asian populations, given that visceral fat is associated with arteriosclerosis and compromised brain health (Debette et al., 2010; Isaac et al., 2011; Kato et al., 2011; Widya et al., 2015).

We also found that depression and osteoporosis increased PD risk, suggesting that depression and osteoporosis preceded the diagnosis of PD. Although depression is a well-known premotor symptom (Tolosa et al., 2007), the temporal relationship between osteoporosis and PD remains poorly understood. Contrary to previous findings of a greater risk of osteoporosis in patients with PD (Torsney et al., 2014), the present study revealed that patients with osteoporosis were at a high risk of PD. Although further studies should be necessary to identify the mechanism, our findings suggested that osteoporosis and PD might have an interactive relationship or that osteoporosis might be a veiled premotor symptom of PD.

Our study has several limitations that should be addressed. First, the discordance between the diagnosis of PD in clinical practice and that recorded in the KNHIS may have led to inaccurate results. However, these issues could be mitigated by the fact that the diagnostic code of PD was classified into the registration code in the program for rare intractable diseases to increase diagnostic accuracy. Additionally, we added the prescription of PD medication for ≥3 months as the outcome definition. Second, we could not define cardiometabolic syndrome using blood pressure measurements and laboratory data such as fasting glucose and total cholesterol levels. Third, we could not assess the exposure time and changes in the risk factors and risk factors that occurred after 2006. Fourth, we could not consider the potential effect of antihypertensive, antidiabetic, and lipid-lowering medication. Fifth, due to a lack of information on a positive family history, traumatic brain injury, exposure to pesticides, dietary patterns, air pollution, and social isolation, we did not identify the PAF of these factors.

Despite the aforementioned limitations, our study aimed to identify age-and sex-specific PD incidence based on a nationwide cohort that included a larger number of elderly participants. In addition, our results highlight the effect of modifiable risk factors for PD in the Korean population, which will help establish health care policies to prevent the development of PD.

## Data availability statement

The datasets presented in this article are not readily available because The Korean NHIS database is confidential and approved for use by researchers who meet the criteria for access through the Korea National Health Insurance Sharing Service (NHISS) Institutional Data Access Committee (https://nhiss.nhis.or.kr/bd/ay/bdaya001iv.do). If data are requested for additional analysis, the corresponding author would consider it deliberately to offer after passing the review process of the Korea NHISS Institutional Data Access Committee and after payment of the data access fee charged to the requester. Requests to access the datasets should be directed to S-BK, parkinson@korea.ac.kr.

## Ethics statement

The studies involving human participants were reviewed and approved by this study was approved by the Institutional Review Board of the Korea University Guro Hospital. Written informed consent for participation was not required for this study in accordance with the national legislation and the institutional requirements.

## Author contributions

SK analyzed and interpreted the data and drafted the manuscript for intellectual content. S-JM and MK analyzed and interpreted the data. SC and GC played major roles in data acquisition. S-BK acquired the data, designed and conceptualized the study, and revised the manuscript for intellectual content. All authors contributed to the article and approved the submitted version.

## Funding

This research was supported by Basic Science Research Program through the National Research Foundation of Korea (NRF) funded by the Ministry of Education (grant number: 2022R1I1A1A01056956) and Korea University Guro Hospital (KOREA RESEARCH-DRIVEN HOSPITAL; grant number: O2208241).

## Conflict of interest

The authors declare that the research was conducted in the absence of any commercial or financial relationships that could be construed as a potential conflict of interest.

## Publisher’s note

All claims expressed in this article are solely those of the authors and do not necessarily represent those of their affiliated organizations, or those of the publisher, the editors and the reviewers. Any product that may be evaluated in this article, or claim that may be made by its manufacturer, is not guaranteed or endorsed by the publisher.

## Footnotes

^1^ http://nhiss.nhis.or.kr

^2^ http://knhanes.kdca.go.kr/
