# Supplementary material for: Incidence of Parkinson’s disease and modifiable risk factors in Korean population: A longitudinal follow-up study of a nationwide cohort
Source: Front Aging Neurosci. 2023 Feb 14;15:1094778. doi: 10.3389/fnagi.2023.1094778 (PMC9971569; doi:10.3389/fnagi.2023.1094778)
Supplement: Supplementary file 1 [file Table_1.DOCX]

|  | Model 1^*^ | | Model 2^#^ | |
| --- | --- | --- | --- | --- |
|  | HR  (95% CI) | *p* | HR^#^  (95% CI) | *p* |
| Hypertension | 1.23 (1.18–1.28) | <0.001 | 1.08 (1.03–1.13) | <0.001 |
| Diabetes | 1.36 (1.29–1.44) | <0.001 | 1.24 (1.17–1.32) | <0.001 |
| Dyslipidemia | 1.33 (1.26–1.40) | <0.001 | 1.14 (1.08–1.21) | <0.001 |
| Ischemic heart disease | 1.35 (1.25–1.46) | <0.001 | 1.13 (1.04–1.22) | <0.001 |
| Depression | 1.79 (1.70–1.89) | <0.001 | 1.68 (1.59–1.77) | <0.001 |
| Osteoporosis | 1.34 (1.28–1.41) | <0.001 | 1.25 (1.19–1.31) | <0.001 |
| Obesity | 1.10 (1.06–1.15) | <0.001 | 1.05 (1.00–1.10) | 0.035 |
| Physical inactivity | 0.99 (0.95–1.04) | 0.680 |  |  |
| Heavy alcohol consumption | 0.97 (0.90–1.04) | 0.397 |  |  |
| Smoking |  |  |  |  |
| Never | Reference |  | Reference |  |
| Ex-smoker | 0.82 (0.75–0.89) | <0.001 | 0.82 (0.75–0.89) | <0.001 |
| Current smoker | 0.79 (0.73–0.84) | <0.001 | 0.81 (0.75–0.86) | <0.001 |

**Supplementary Table 1.** Hazard ratio of modifiable risk factors for PD in the participants without stroke

^*^Model 1: Each risk factor was entered as a predictor in the Cox regression model after controlling for age and sex.

^#^Model 2: All risk factors associated with PD in model 1 were included as predictors after controlling for age and sex.

Abbreviations: HR, hazard Ratio; PD, Parkinson’s disease
